# Survival and Complication Rates of Ceramic Partial Coverage Restorations (PCRs) and Ceramic Laminate Veneers Made of Different Types of Ceramics. Consensus Statement From SSRD, SEPES, and PROSEC Conference on Minimally Invasive Restorations

**DOI:** 10.1111/jerd.13418

**Published:** 2025-04-17

**Authors:** Florian Beuer, Stefano Pieralli, Frank Spitznagel, Dubravka Knezović Zlatarić, Hannah Bleiel, Petra Gierthmühlen, Sigmar Schnutenhaus, Jörg Strub, Luc Rutten, P. Rutten, Marco Jäggi, Burak Yilmaz, José Manuel Reuss, Ernest Mallat‐Callis, Jaime Jiménez, Rafael Martinez‐de‐Fuentes, Miguel A. Ortiz, Miguel Gómez‐Polo, Patrick Klein

**Affiliations:** ^1^ Charité – Universitätsmedizin Berlin, Zahnärztliche Prothetik, Alterszahnmedizin und Funktionslehre Berlin Germany; ^2^ Charité – Universitätsmedizin Berlin Charité Centrum 3 für Zahn‐, Mund‐ und Kieferheilkunde Zahnärztliche Prothetik, Alterszahnmedizin und Funktionslehre Berlin Germany; ^3^ Universitätsklinikum Düsseldorf, Poliklinik für Zahnärztliche Prothetik, Leitender Oberarzt, Poliklinik für Zahnärztliche Prothetik Düsseldorf Germany; ^4^ Dept of Prosthodontics, School of Dental Medicine University of Zagreb Zagreb Croatia; ^5^ Charité – Universitätsmedizin Berlin, Abteilung für Zahnärztliche Prothetik, Alterszahnmedizin und Funktionslehre Berlin Germany; ^6^ Universitätsklinikum Düsseldorf, Poliklinik für Zahnärztliche Prothetik Düsseldorf Germany; ^7^ Zentrum für Zahnmedizin MVZ GmbH Hilzingen Germany; ^8^ Department of Restorative Dentistry University Freiburg Freiburg Germany; ^9^ Dental Team bv Tessenderlo Belgien; ^10^ Clinic for Reconstructive Dentistry University Center for Dentistry Basel UZB Basel Switzerland; ^11^ Departments of Reconstructive Dentistry and Gerodontology, and Restorative, Preventive, and Pediatric Dentistry University of Bern Bern Switzerland; ^12^ Odontologo Especialista en Cirugia Oral Clinica Vilaboa, Profesor Colaborador Departamento de Prótesis Universidad Complutense y asignatura Estética Dental de Universidad San Pablo CEU Madrid Spain; ^13^ Director Postgraduate Program in Prosthodontics and Oral Rehabilitation (SCOE) Barcelona Spain; ^14^ Chairman Implant Department Universidad Europea de Madrid (UEM) Madrid Spain; ^15^ University of Seville, Faculty of Dentistry Seville Spain; ^16^ DentLit LLC 18 Rockland St. Newton Heights, MA USA; ^17^ Profesor Titular de Universidad Associate Professor School of Dentistry Complutense University of Madrid Madrid Spain; ^18^ Universitätsklinikum Düsseldorf, Zahnarzt, Wissenschaftlicher Mitarbeiter DGI‐zertifizierter Implantologe, Studienleiter für klinische Studien Düsseldorf Germany

**Keywords:** ceramic laminate veneers, clinical research, clinical trials, material sciences, partial coverage restorations

## Abstract

**Objectives:**

This consensus paper aims to provide evidence‐based insights into the survival and complication rates of ceramic partial coverage restorations (PCRs) and ceramic laminate veneers, focusing on various ceramic materials, including feldspathic ceramics, leucite‐reinforced glass ceramics (LRGC), lithium disilicate ceramics (LDS), and resin matrix ceramics (RMC).

**Material and Methods:**

A systematic screening of the literature identified 35 publications, which were critically reviewed based on PRISMA guidelines, resulting in two systematic reviews. Consensus statements were formulated from the findings of these reviews, addressing key clinical and material‐specific considerations. These statements were subsequently refined and finalized through expert discussion, ensuring alignment with the current evidence base and clinical applicability.

**Results:**

PCRs demonstrated high short‐term survival rates, with 93.7% for LDS and 89.3% for RMC. LDS showed slightly better performance in terms of retention and fracture resistance. Ceramic laminate veneers exhibited excellent long‐term survival rates across all materials, with feldspathic veneers at 96.13%, LRGC at 93.7%, and LDS at 96.81%. LDS ceramic veneers showed a significantly lower complication rate compared to feldspathic and LRGC veneers.

**Conclusions:**

Both PCRs and ceramic laminate veneers are reliable treatment options, offering high survival rates and manageable complication profiles. Material selection and minimally invasive techniques are key to optimizing clinical outcomes. Further research is needed to implement standardized protocols for preparation, adhesive techniques, and long‐term maintenance.

## 1 Introduction

Advancements in adhesive dentistry and ceramic materials have redefined restorative dental practices, providing clinicians’ innovative approaches to manage complex cases. Ceramic partial coverage restorations (PCRs) and laminate veneers have emerged as key solutions for addressing both esthetic and functional challenges. These restorations, grounded in minimally invasive principles, prioritize tooth preservation while meeting patients' expectations for durability and esthetics. However, their success relies on a comprehensive understanding of the clinical performance of various ceramic materials and the implementation of evidence‐based protocols.

Recognizing the significance of these considerations, Group 1 of the Joint SSRD, SEPES, and PROSEC Consensus Conference provided clinical statements and recommendations for PCRs and laminate veneers.

Prior to the Consensus Conference, two systematic reviews were prepared and reviewed by Working Group 1. The first review [1] examined the performance of PCRs for posterior teeth. These restorations, including onlays, occlusal veneers, and partial crowns, offer a minimally invasive alternative to full crowns, preserving as much of the natural tooth structure as possible. Materials such as lithium disilicate (LDS) and resin matrix ceramics (RMC) were analyzed, focusing on their survival rates, mechanical resistance, and susceptibility to technical or biological complications. PCRs play a pivotal role in restorative dentistry, especially for patients with extended posterior defects or compromised occlusal stability.

The second review [2] investigated the survival and complication rates of ceramic laminate veneers, focusing on materials, including feldspathic ceramics, leucite‐reinforced glass ceramics (LRGC), LDS, and zirconia. These veneers, applied for anterior teeth, combine excellent esthetic outcomes with the benefits of minimally invasive preparation techniques. Despite their advantages, challenges such as debonding, marginal discoloration, and fracture risks highlight the need for a comprehensive understanding of material‐specific behavior and long‐term outcomes.

Based on the data and meta‐analyses of these systematic reviews and thorough discussions among the participants of Group 1 and the Consensus Conference plenary, clinical recommendations were carefully formulated, providing guidance on material selection, preparation design, and long‐term maintenance strategies. Additionally, the paper identifies areas with limited evidence, emphasizing the need for further research to refine clinical protocols and enhance patient outcomes. Ultimately, this work aims to support clinicians in achieving predictable and durable results while advancing the field of adhesive and restorative dentistry.

## 2 Systematic Review Paper 1

### 2.1 Manuscript Title

Survival and Complications of Partial Coverage Restorations on Posterior Teeth—A Systematic Review and Meta‐Analysis.

### 2.2 Major Findings of the Review

#### 2.2.1 Major Findings of the Review 1

PCRs and RMC showed similar (not significantly different) survival rates after 3 years of clinical service time: 93.7% and 89.3%, respectively.

### 2.3 Major Answered Questions

#### 2.3.1 Major Answered Question 1

LDS and RMC showed similar results in terms of failures per 100 restoration‐years (2.13 for LDS; 3.69 for RMC), chipping (1.89 for LDS; 2.29 for RMC), loss of retention (0.98 for LDS; 2.76 for RMC), endodontic complications (0.53 for LDS; 0.92 for RMC), and bulk fractures (1.07 for LDS; 0.32 for RMC). The analysis is based on a follow‐up period of 1 to 3 years, 252 restorations (4 RCTs), except for loss of retention, which is based on 2 to 3 years, 212 restorations (3 RCTs).

### 2.4 Major Unanswered Questions

#### 2.4.1 Major Unanswered Question 1

There are few studies referred to the original research except for: LDS, RMC, and LRGC.

#### 2.4.2 Major Unanswered Question 2

There is little long‐term data in the original research on survival rates of LDS, RMC, and LRGC included in the meta‐analysis.

### 2.5 Clinical Recommendations

#### 2.5.1 Clinical Recommendation 1

Based on the RCTs of the meta‐analysis and clinical data from RCTs included in the systematic review, PCRs from RMC, LDS, and LRGC can be considered a reliable option (short to mid‐term [7 years]).

#### 2.5.2 Clinical Recommendation 2

Clinicians need to be careful about controlling multiple risk factors, which contribute to the wide variability observed in reported survival rates.

### 2.6 Recommendations for Future Research

#### 2.6.1 Recommendations for Future Research 1

Including the state of the tooth (restorative history of the tooth, the amount and integrity of tooth structure) before new treatment approach.

#### 2.6.2 Recommendations for Future Research 2

Monitoring of the integrity of the restoration‐tooth‐complex and the opposing dentition.

#### 2.6.3 Recommendations for Future Research 3

Incorporating promising new materials and approaches that have advanced beyond in vitro testing.

#### 2.6.4 Recommendations for Future Research 4

Identifying a standardized adhesive approach for each material.

#### 2.6.5 Recommendations for Future Research 5

RCTs including the use of split‐mouth designs and larger sample sizes with longer follow‐ups.

#### 2.6.6 Recommendations for Future Research 6

Standardized assessment methods across clinical outcomes.

### 2.7 Consensus Statement

#### 2.7.1 Consensus Statement 1

Based on the data, all included materials (LDS, RMC, and LRGC) seemed to be appropriate treatment options for PCRs in the posterior region in the mid‐term.

#### 2.7.2 Consensus Statement 2

Emphasizing tooth preservation over restoration durability, minimally invasive techniques should be prioritized, transferring potential risks from the remaining tooth structure to the prosthetic restoration.

#### 2.7.3 Consensus Statement 3

Focus should be on the longevity of the tooth.

#### 2.7.4 Consensus Statement 4

Repairability and the maintenance of the restoration should be considered prior to replacement.

## 3 Systematic Review Paper 2

### 3.1 Manuscript Title

Survival and Complication Rates of Feldspathic, Leucite‐Reinforced, Lithium Disilicate and Zirconia Ceramic Laminate Veneers: A Systematic Review and Meta‐Analysis.

### 3.2 Major Findings of the Review

#### 3.2.1 Major Findings of the Review 1

Ceramic laminate veneers showed high survival rates across all time intervals (2.6Y: 97.76%; 5.0Y: 97.12%, 10.4Y: 96.05%). The long‐term evaluation of different ceramic materials yielded:

- Feldspathic porcelain: 96.13%.
- Leucite‐reinforced glass ceramics (LRGC): 93.7%.
- Lithium disilicate (LDS): 96.81%.

#### 3.2.2 Major Findings of the Review 2

LDS showed significantly better results compared to feldspathic (*p* = 0.044) and LRGC for laminate veneers (*p* = 0.033) in terms of technical and biological complications.

#### 3.2.3 Major Findings of the Review 3

No significant difference was found for pooled esthetic complications between different ceramic materials at long‐term follow‐up (feldspathic vs. LRGC *p* = 0.972, feldspathic vs. LDS *p* = 0.117 and LRGC vs. LDS *p* = 0.087).

### 3.3 Major Answered Questions

#### 3.3.1 Major Answered Question 1

Ceramic laminate veneers fabricated from feldspathic, LRGC, and LDS are a reliable treatment option with favorable long‐term results for the restorations.

#### 3.3.2 Major Ans26wered Question 2

Complications arise over time and affect the longevity of ceramic laminate veneer‐tooth complex.

### 3.4 Major Unanswered Questions

#### 3.4.1 Major Unanswered Question 1

No clear statement about the preferable fabrication method of the ceramic laminate veneer can be made (conventional vs. CAD/CAM).

#### 3.4.2 Major Unanswered Question 2

There is no definitive guidance on the bonding protocol and adhesive cementation.

#### 3.4.3 Major Unanswered Question 3

The indication of ceramic laminate veneers, the preparation design (extension), the necessity (prep vs. non‐prep), and the amount of tooth structure removal are unknown.

#### 3.4.4 Major Unanswered Question 4

The mid‐ and long‐term performance of zirconia laminate veneers remains uncertain.

#### 3.4.5 Major Unanswered Question 5

Limited information is given about the thickness and design (monolithic vs. veneered) of the ceramic laminate veneers.

### 3.5 Clinical Recommendations

#### 3.5.1 Clinical Recommendation 1

Feldspathic, LRGC, and LDS laminate veneers with different clinical and laboratory protocols reveal high long‐term survival rates and can be recommended.

#### 3.5.2 Clinical Recommendation 2

Ceramic laminate veneers should ideally be bonded to enamel, since bonding to larger areas of dentin and/or existing restorations seem to be more likely to cause complications over time.

#### 3.5.3 Clinical Recommendation 3

Clinicians should determine in advance whether a preparation is required and if needed, flat incisal overlapping (butt‐joint), might be preferred, as it showed lower failure rates for the restorations compared to non‐overlapping or palatal chamfer.

### 3.6 Recommendations for Future Research

#### 3.6.1 Recommendations for Future Research 1

Encourage a thorough evaluation of the indication criteria for veneers.

#### 3.6.2 Recommendations for Future Research 2

Need of RCTs on different workflows (analog and digital), materials, and adhesive protocols (material tooth substrate combination) evaluated as a restoration‐tooth‐complex.

#### 3.6.3 Recommendations for Future Research 3

RCTs including the use of split‐mouth designs and larger sample sizes with longer follow‐ups.

#### 3.6.4 Recommendations for Future Research 4

Exploration of the potential of different zirconia materials for laminate veneers.

#### 3.6.5 Recommendations for Future Research 5

Assessment of preparation design, thickness, and extension of ceramic laminate veneers.

#### 3.6.6 Recommendations for Future Research 6

Evaluation of monolithic versus layered laminate veneers.

#### 3.6.7 Recommendations for Future Research 7

Analysis of patient‐reported outcome measurements (patient‐reported outcome measures, e.g., dental impact on daily living) and development of clinician‐reported outcome criteria.

#### 3.6.8 Recommendations for Future Research 8

Monitoring of the restorations to detect any kind of changes over time and application of existing scores (FDI‐criteria).

### 3.7 Consensus Statement

#### 3.7.1 Consensus Statement 1

Based on the data, all included materials (feldspathic porcelain, LRGC, and LDS) seemed to be appropriate options for laminate veneers.

#### 3.7.2 Consensus Statement 2

The indication of veneers should be made under strict ethical considerations of preserving sound tooth structure and least‐invasive treatment options should be considered first.

#### 3.7.3 Consensus Statement 3

A comprehensive understanding—from diagnostic to preparation to precise adhesive cementation—is essential for the successful, long‐term outcomes of ceramic laminate veneers.

#### 3.7.4 Consensus Statement 4

To enhance the longevity of ceramic laminate veneers, clinicians should recommend the following protective measures:

- Regular recall appointments.
- Occlusal splints (in patients with parafunctional habits).

## Conflicts of Interest

The authors declare no conflicts of interest.

## Data Availability

Data sharing not applicable to this article as no datasets were generated or analysed during the current study.
